# Nature and outcome of malignant goiter: a revisit

**DOI:** 10.11604/pamj.2021.38.204.27503

**Published:** 2021-02-23

**Authors:** Mohamed Yasser Ibrahim Daoud

**Affiliations:** 1 Division of General Surgery, Department of Surgery, College of Medicine, King Faisal University, Riyadh, Kingdom of Saudi Arabia

**Keywords:** Goiter, malignancy, follicular carcinoma, papillary carcinoma, lymphoma

## Abstract

**Introduction:**

the aim of this retrospective study is to review patients with malignant goiter, as regards their nature, demographic characterization, clinical presentation and preoperative histopathological data.

**Methods:**

the study focused on a period of 4 years from December 2015 to January 2019. Patients´ demographic data, clinical presentation, Intra-operative findings, Pre and postoperative histopathological staging and grading were all recorded. Postoperative follow up whether early or late were also included.

**Results:**

a total of 100% (n = 65) patients underwent surgery. The female to male ratio was found to be 5:1 (48 females and 17 males). Solitary nodule was the main clinical presentation in 80% (n = 52) of cases while 20% (n = 13) were multinodular swellings. Papillary carcinoma was recorded in 80% (n = 52) of patients while in 15.4% (n =10) of patients were having follicular carcinoma. The remaining 4.6% (n = 3) suffered of lymphoma; no medullary or anaplastic tumors were reported.

**Conclusion:**

thyroid cancer is the most commonly encountered endocrinal malignancy at our institute. Fine Needle Aspiration Cytology (FNAC) showed a high percentage of reliability in diagnosing thyroid cancer among our series. It is recommended to adapt this technique in initial screening of goiter patients in our local setting.

## Introduction

Thyroid cancer is the commonest malignant tumor of the endocrine system [1]. In the kingdom of Saudi Arabia, it constitutes 8.8% of malignancies. Moreover, thyroid cancer is reported to be the second commonest female cancer (12%) nationwide. Nevertheless, it has a higher incidence among females than males with a ratio of 1 to 0.3 [2,3]. Conclusively, the overall incidence of the newly diagnosed thyroid cancer accounts for 5.4% of all newly diagnosed cancer within the country [4,5].

In 2008, the Saudi Cancer Registry ranked thyroid cancer as the second commonest malignancy in women while in men it is the thirteenth. The areas that have the higher incidences were as follow; Tabuk (6/100,000), Eastern province (5.9/100,000), Riyadh (5.8/100,000), Qassim (5.7/100,000) and the northern region (4.9/100,000). Thyroid cancer occurs at a relatively earlier age with median of 40 and 44 years for females and males respectively. The commonest histological type was papillary adenocarcinoma [6].

However, thyroid cancer ranks the fifth commonest malignancy among the Gulf Cooperation Council (GCC) countries with a higher incidence among females. It stands as the second commonest female malignancy in GCC [7]. They may present as thyroid nodule or even multinodular goiter with some extra glandular manifestations of compression such as dysphagia and/or dyspnea. Sometimes patient may present with thyrotoxicosis manifestations. Yet, in most cases, they present as asymptomatic nodules in euthyroid patients [8-10].

Thyroid cancer treatment entails many modalities such as radical thyroidectomy, radioactive ablation and hormonal replacement therapy to antagonize thyroid-stimulating hormone. Recently new therapeutic modalities have been invented concentrating on specific mutation pathways and intranuclear gene regulation [11]. The current study took place in a tertiary care hospital in the Eastern Province of Saudi Arabia, aiming to review malignant goiter patients with regards to their nature, demographic characterization, clinical presentation and preoperative histopathological data obtained by Fine Needle Aspiration Cytology (FNAC). It also aimed to review the operative data and post-operative histopathological pattern of all patients. Moreover, the study was designed to evaluate the nature, line of management and outcomes of malignant goiter in the same geographical region.

## Methods

**Study setting and design:** this descriptive retrospective cohort study was carried out during the period between December 2015 to January 2019.

**Study population:** all patients with malignant goiter at different age groups, who were admitted and treated during this four-year period.

**Data collection:** patients´ records were thoroughly reviewed to report their demographic data such as; age, gender, nationality, place of residence and occupation. Also reported, were the clinical presentation including the nature of enlargement, presence of toxic symptoms, results of FNAC and co-morbid conditions. Operative findings, presence of lymph node enlargement, Tumor, Nodes, Metastasis (TNM) staging and the implemented surgical technique were also retrieved from the files. The pre- and post-operative microscopic pathology, type of malignancy, pathological staging, histological grading, and vascular involvement were recorded. Lengths of postoperative follow up, secondary surgical procedures or radioiodine treatment were looked for.

**Statistical analysis:** data were expressed in numbers and percentages as well as mean and standard deviation for analytic purposes.

**Ethical approval and consent to participate:** experiments were conducted after obtaining the institutional researchers' board (IRB) of college of Medicine, King Faisal University Ethical approval number 33-8-29RSR on the manuscript.

## Results

**Sociodemographic data:** one hundred percent (100%) (n = 65) of patients were evaluated during the four-year study period. Saudi nationals were 93.8% (n = 61).The female to male ratio was 5: 1 (54 females, 11 males). The mean age was 34.4±11.7 (mean±SD). The youngest was an 8-year-old boy while the eldest was a 70-year-old female with multinodular goiter. Almost two thirds (62%) of patients were between 21 years and 40 years (Table 1). All patients were originally from AlAhsa, and were living there without any predilection to any specific village or town. Forty percent (26/65) of malignancies were from the northern part of Al-Ahsa, with a significant percentage from Al-Omran village.

**Table 1:** age distribution of the studied sample

Age group	Number (Percentage)
Less than 20 years	10.7% (n = 7)
21-30 years	32.3% (n = 21)
31-40 years	29.3% (n = 19)
41-50 years	13.9% (n = 9)
51-60 years	7.7% (n = 5)
61-70 years	6.1% (n = 4)

**Investigations results:** FNAC initially showed 87.7% (n = 57) to be malignant (Bethesda category VI) [12]. Yet, 7.7% (n = 5) were suspicious for malignancy (Bethesda category V) and 4.6% (n = 3) showed atypia (Bethesda category III). Most cases were of the euthyroid status except for 13.8% (n = 9) who showed hyperthyroidism. Solitary thyroid nodules (STN) were the most common presentation among the whole 100% (n = 65) studied patients with a percentage of 80% (n = 52) while the remaining 20% (n = 13) had multinodular swellings. All patients (n = 65) underwent post-operative histopathological assessment that proved malignancy despite the initial FNAC results.

**Figure 1:**
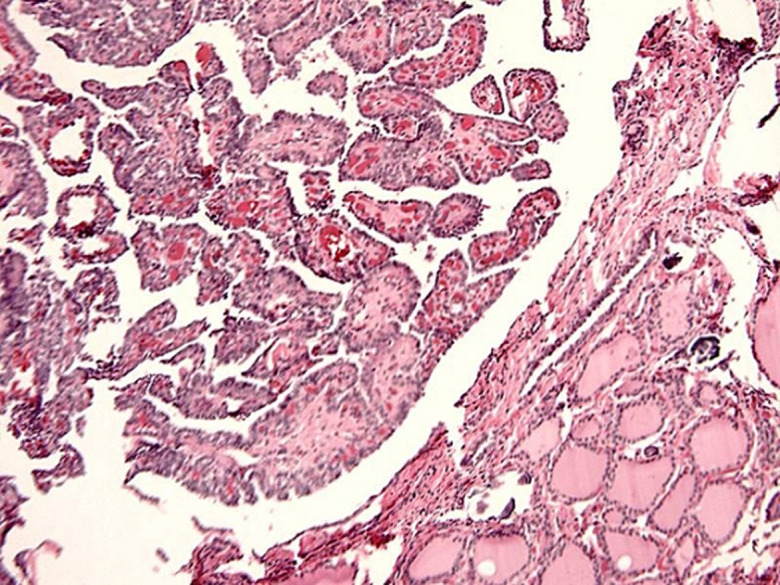
post-operative histopathology papillary carcinoma

**Figure 2:**
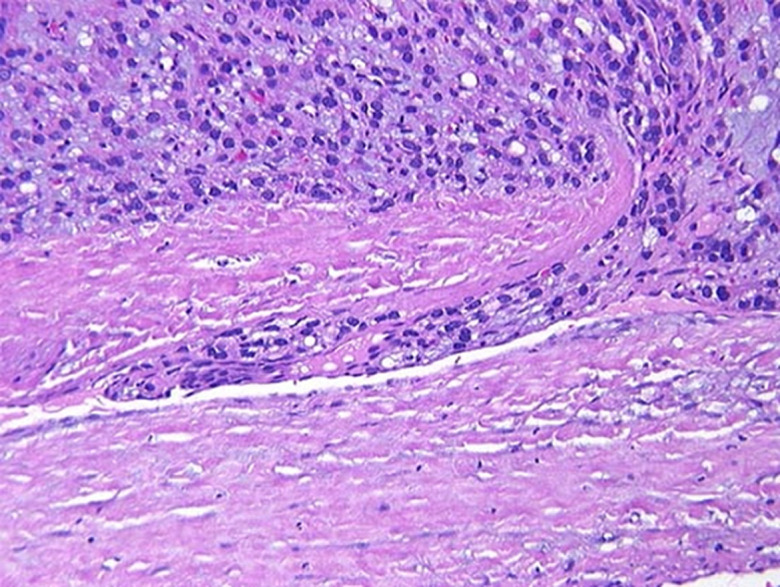
post-operative histopathology follicular carcinoma

**Figure 3:**
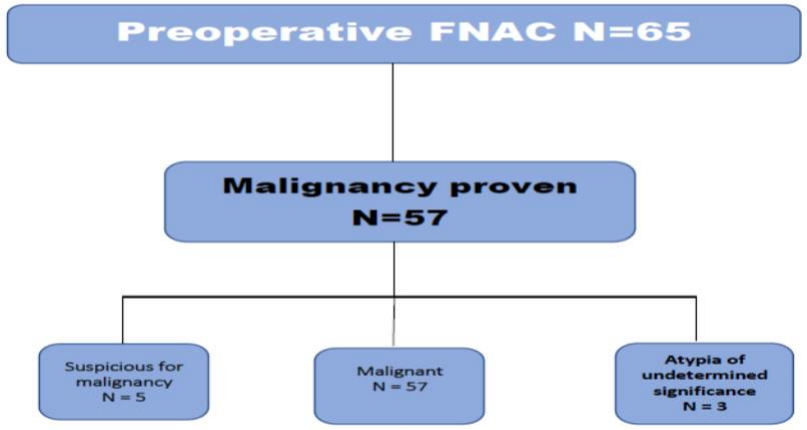
preoperative fine needle aspiration cytology (FNAC) chart

**Figure 4:**
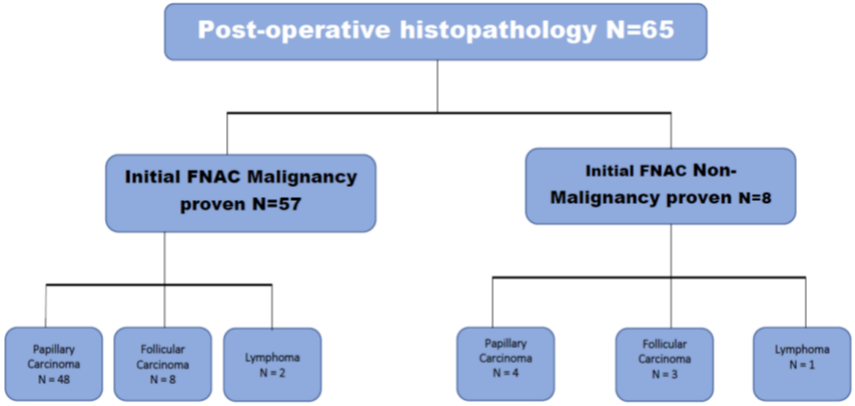
post-operative histopathology

**Operative and post-operative findings:** post-operative findings showed papillary carcinoma to be found in 80% (n = 52) (Figure 1), follicular carcinoma in 15.4% (n = 10) (Figure 2) and lymphoma in 4.6% (n = 3). There were no medullary or anaplastic tumors. Table 2 showed the international tumor-node-metastasis (TNM) of the samples. Intraoperatively, those patients who showed evidence of lymph node involvement were treated by systematic compartment orientated selective nodal dissection. Most of them were done through extended thyroid incision or even Hshaped. Excision of affected lymph nodes was performed in all the thirteen patients. The mean follow-up period was 14 months with a range from one up to 45 months. A little more than fifty two percent (52.3%) (n = 34) had a follow up for one year, 32.3% (n = 21) between one and three years, 13.8% (n = 9) between three and for years and one was followed up for five years. The pre- and post-operative FNAC results are shown in Figure 3, Figure 4 respectively.

**Table 2:** tumor node metastasis (TNM) staging

Tx	T1	T2	T3	T4	N 1 a Level VI	N 1 a Level VII	N 1 b	M0
7.7% (n = 5)	63% (n= 41)	13.8% (n = 9)	15.3% (n = 10)	00	7.7% (n = 5)	3% (n = 2)	9.2% (n = 6)	00

Tx: primary tumor cannot be assessed. T1: tumor ≤ 2 cm in greatest dimension limited to the thyroid. T2: tumor > 2cm but ≤ 4cm in greatest dimension limited to the thyroid. T3: tumor > 2cm limited to the thyroid. T4: gross extrathyroidal extension. N 1a level VI: Metastasis to pretracheal lymph nodes. N 1a level VII: Metastasis to prelaryngeal lymph nodes. N 1b: Metastasis to level I, II, III lymph nodes. M 0: No distant metastasis

## Discussion

Thyroid disorders constitute one of the common chronic diseases. In a study of family practice in Saudi Arabia, it was seen that 3.5% of patients attending the family health clinic suffered from thyroid disorders [13]. Thyroid cancer is considered the commonest endocrinal malignancy with variable incidence from a country to another. However, the incidence of thyroid malignancies is still increasing with many characteristic changes. In our current retrospective study, papillary carcinoma was the highly predominant thyroid cancer, while follicular carcinoma and lymphoma were less frequent. These data coincide with previously published reports from the same country [14,15]. A study reported papillary carcinoma to have an incidence of 82.2% and follicular carcinoma to be 4.4% among thyroid malignancies. This report supports our current data, although it showed a lower frequency in incidence of follicular cancer [15,16] (4.4%) compared to 8.3% follicular thyroid cancer in the current study [15]. Yet, it was coinciding with other reports from the Kingdom of Saudi Arabia [16,17]. Etiological factors for thyroid malignancy such as familial tendency, endemic goiter and exposure to irradiation were not reported in any our patients. The current study showed Females to have a higher prevalence of thyroid malignancies compared to their male counterparts with the ratio of 5: 1. These data were similar to other gathered in the same country [18].

However, others showed male predominance among thyroid cancer patients. They attributed this finding to the ethnic background of the patients who were not Saudi in comparison to the Saudi population. This may be explained by the demographic pattern of the expatriates in Saudi Arabia. In fact, they were mostly men. Therefore, this incidence, due to sampling bias, should be cautiously interpreted [19,20]. We have reported the mean patients´ age to be 34.4 years. This is relatively lower than other local and international studies [18-20]. It may be explained by the changes of the clinical and epidemiological behavior of the disease worldwide [21]. Some reports denoted that thyroid cancer patients´ age may influence prognosis of the disease beside other factors such as; the histopathological findings, tumor size, local extension, nodal status and the operative procedure. Meta analytic studies denoted that distant metastasis, age and tumor size are highly significant prognostic factors [22,23]. On the other hand, others related the prognostic effect of age on the outcome of thyroid cancer patients to the higher prevalence of associated pathologies among older patients [24,25]. We reported solitary thyroid nodule to be the most common presentation in our series of thyroid malignancy in accordance with data in the literature [26].

Others reported contradictory data, where cases of multinodular goiter (66.7%) presented with thyroid cancer due to the late presentation among their patients [18]. FNAC confirmed the diagnosis of thyroid cancer in 88% of patients. This was similar to previously published data [18,27]. Yet, those patients with negative FNAC proved to have thyroid cancer in the post-operative histopathological specimen. Due to the limited sample size as data were collected only from the Eastern Province of Saudi Arabia. Further studies and metanalytic reports need to be addressed to conclude a solid holistic view of the problem of thyroid cancer.

## Conclusion

Thyroid cancer is the most commonly frequent endocrinal malignancy in our institute as well as worldwide. FNAC showed a high positive percentage of reliability for diagnosing thyroid cancer. Therefore, it is recommended to be adopted as the initial tool of screening goiter patients.

### What is known about this topic

- Thyroid cancer is the commonest malignant tumor of the endocrine system;
- In the kingdom of Saudi Arabia, it constitutes 8.8% of malignancies;
- Thyroid cancer ranks the fifth commonest malignancy among the Gulf Cooperation Council (GCC) countries.

### What this study adds

- Malignant goiter is increasing in the eastern province of Saudi Arabia;
- Papillary carcinoma is the mostly commonly encountered cancer among the studied cohort (80%);
- FNAC is reliable in almost 88% of cases in relation to the post histopathological results.
